# Supplementary figures and images for: Cell-surface tethered promiscuous biotinylators enable comparative small-scale surface proteomic analysis of human extracellular vesicles and cells
Source: eLife. 2022 Mar 8;11:e73982. doi: 10.7554/eLife.73982 (PMC8983049; doi:10.7554/eLife.73982)

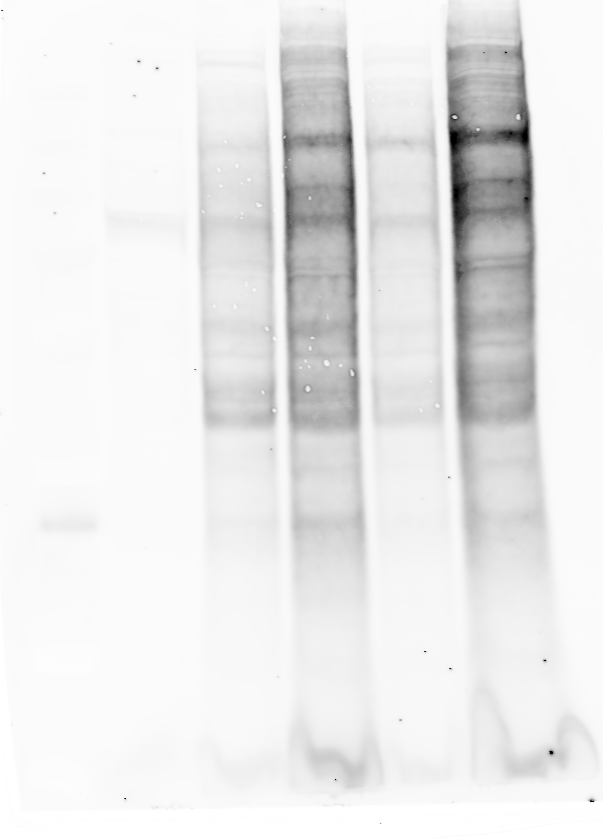

Supplement: Figure 2—source data 1. [file elife-73982-fig2-data1.zip › Figure2-Source data 1_Streptavidin (uncropped).png]

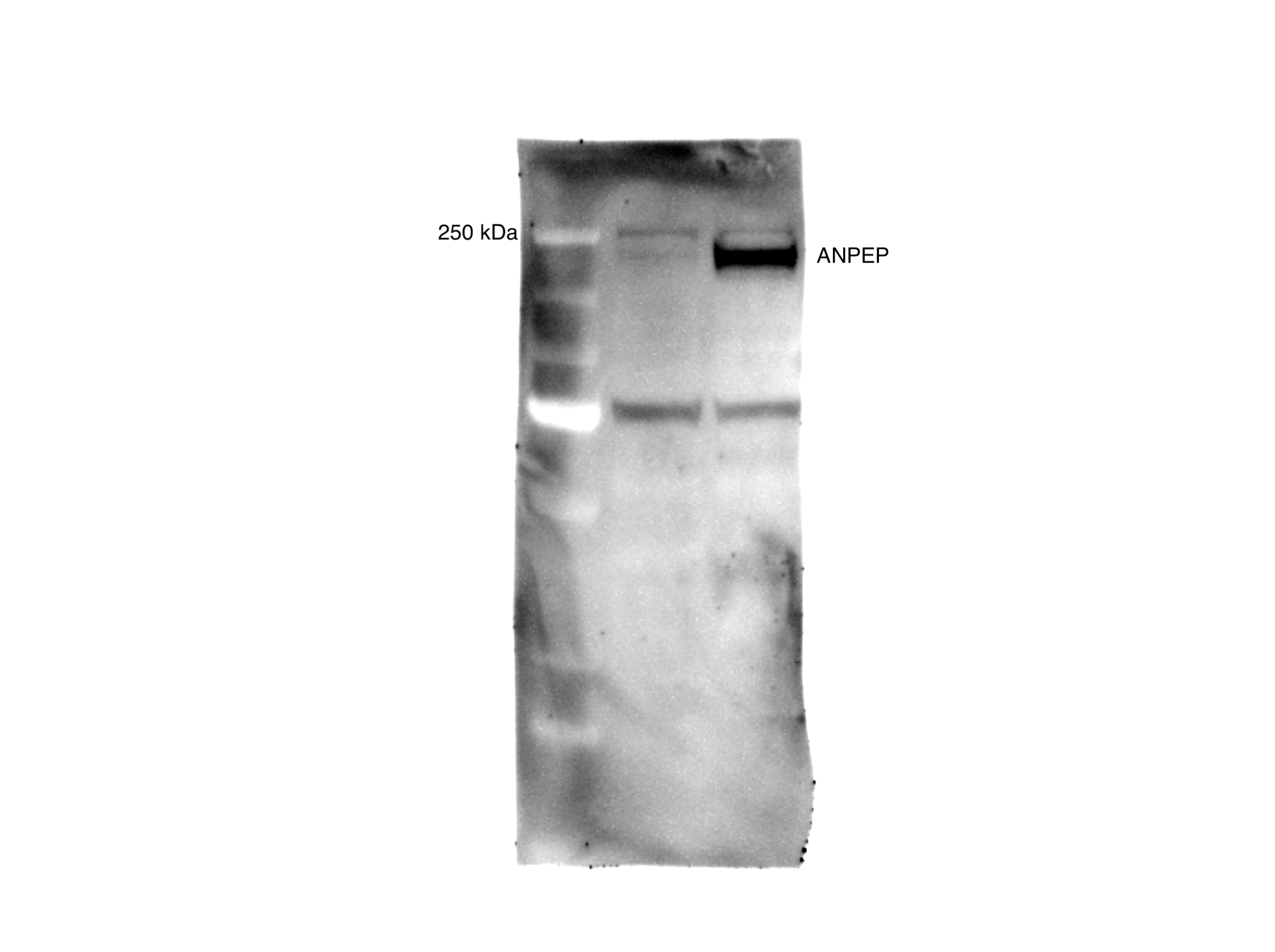

Supplement: Figure 3—source data 1. [file elife-73982-fig3-data1.png]

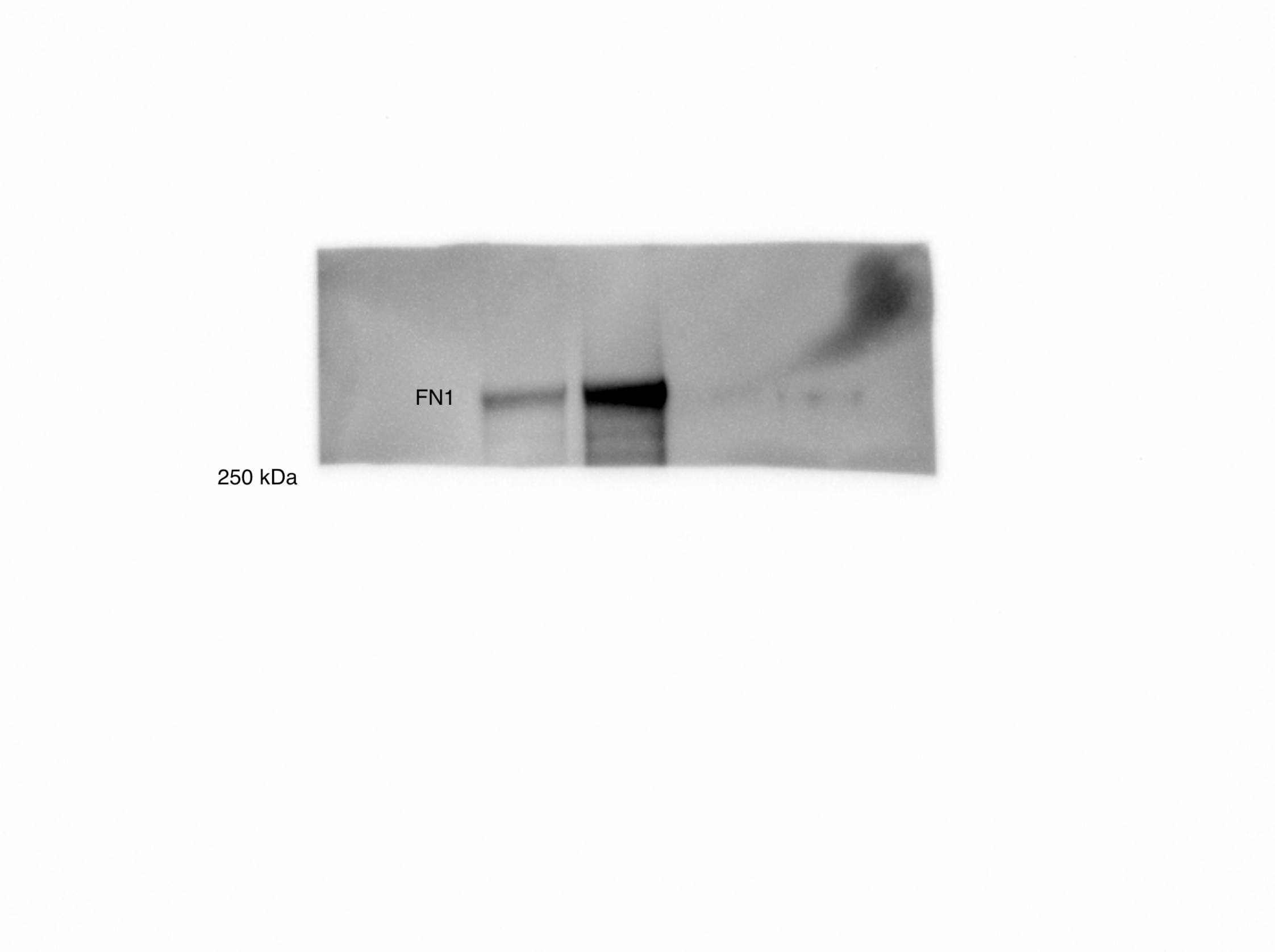

Supplement: Figure 4—source data 1. [file elife-73982-fig4-data1.png]

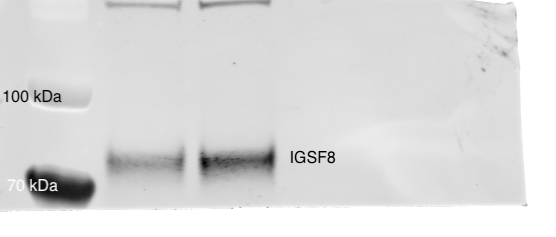

Supplement: Figure 5—source data 1. [file elife-73982-fig5-data1.png]

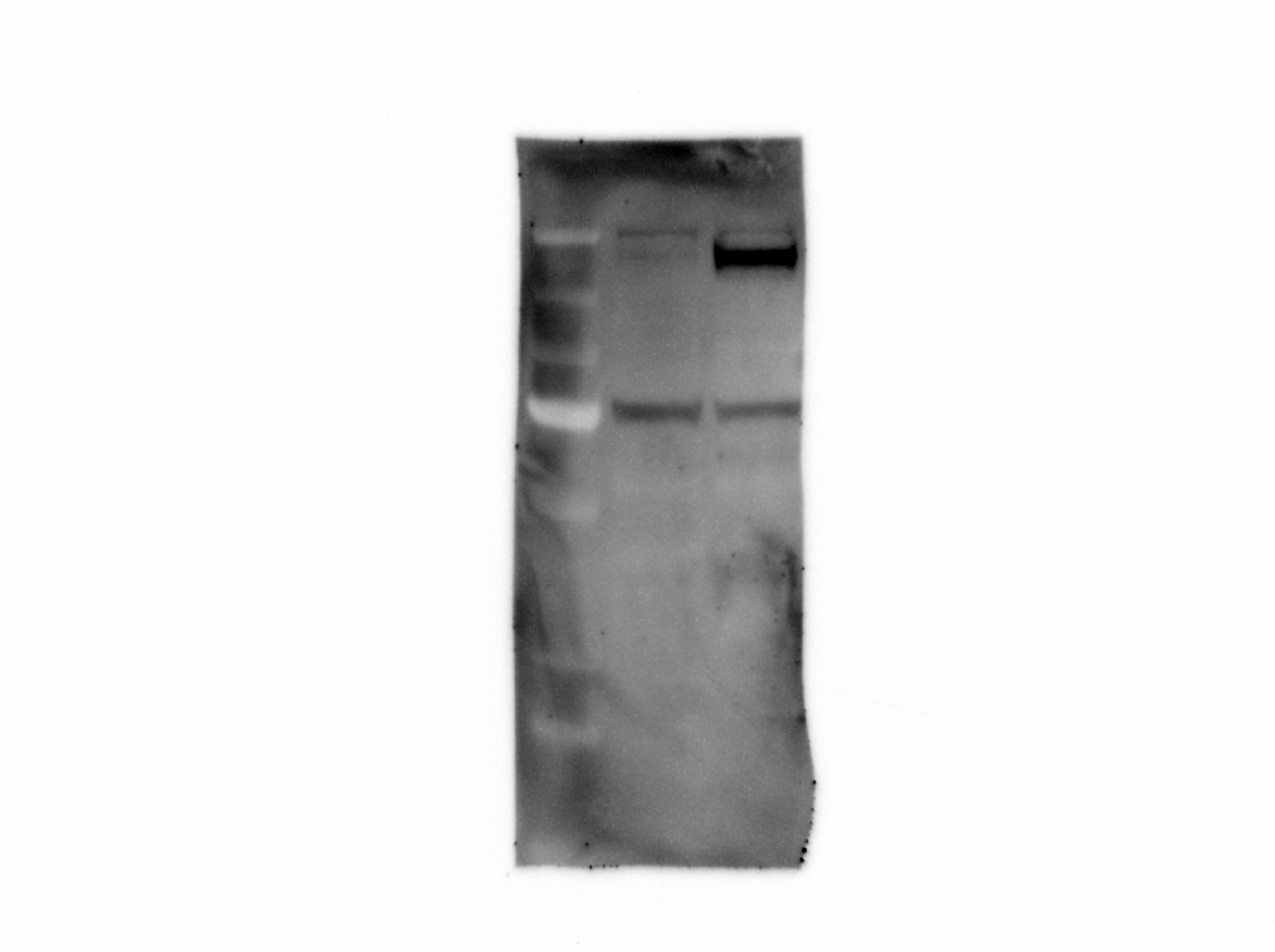

Supplement: Source data 1. [file elife-73982-data1.zip › WesternBlots/Figure3-Source data_1_ANPEP (raw).tif]

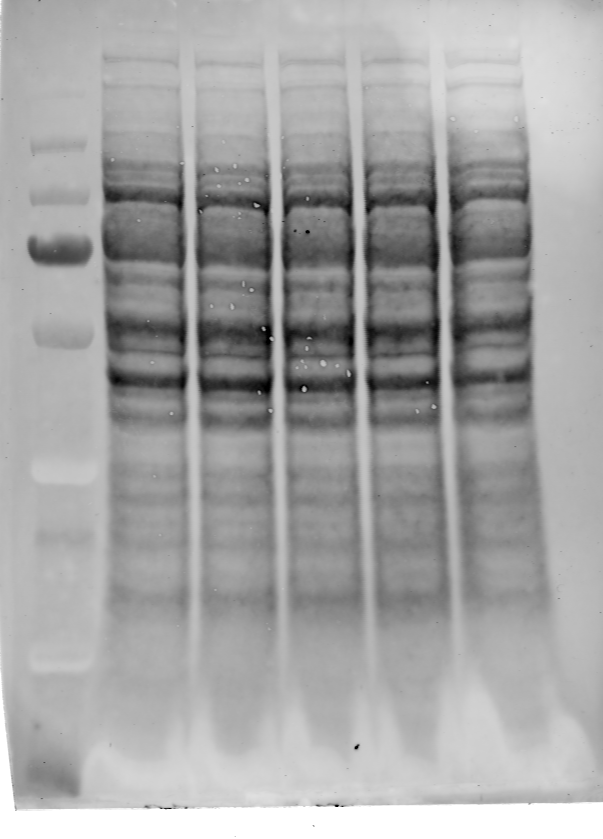

Supplement: Source data 1. [file elife-73982-data1.zip › WesternBlots/Figure2-Source data 1_TotalProtein (uncropped).png]

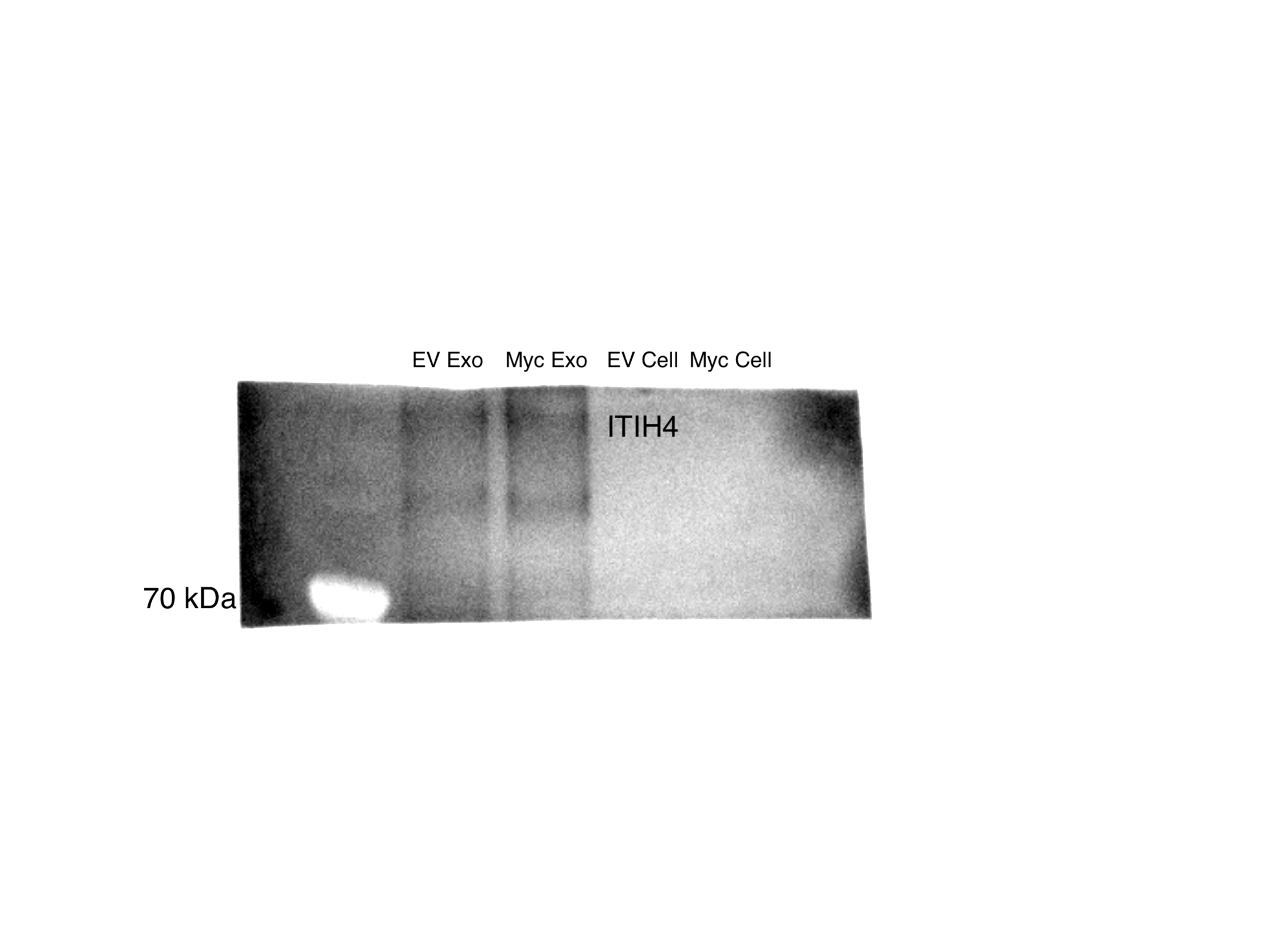

Supplement: Source data 1. [file elife-73982-data1.zip › WesternBlots/Figure5-Source data 1_ITIH4 (uncropped).png]

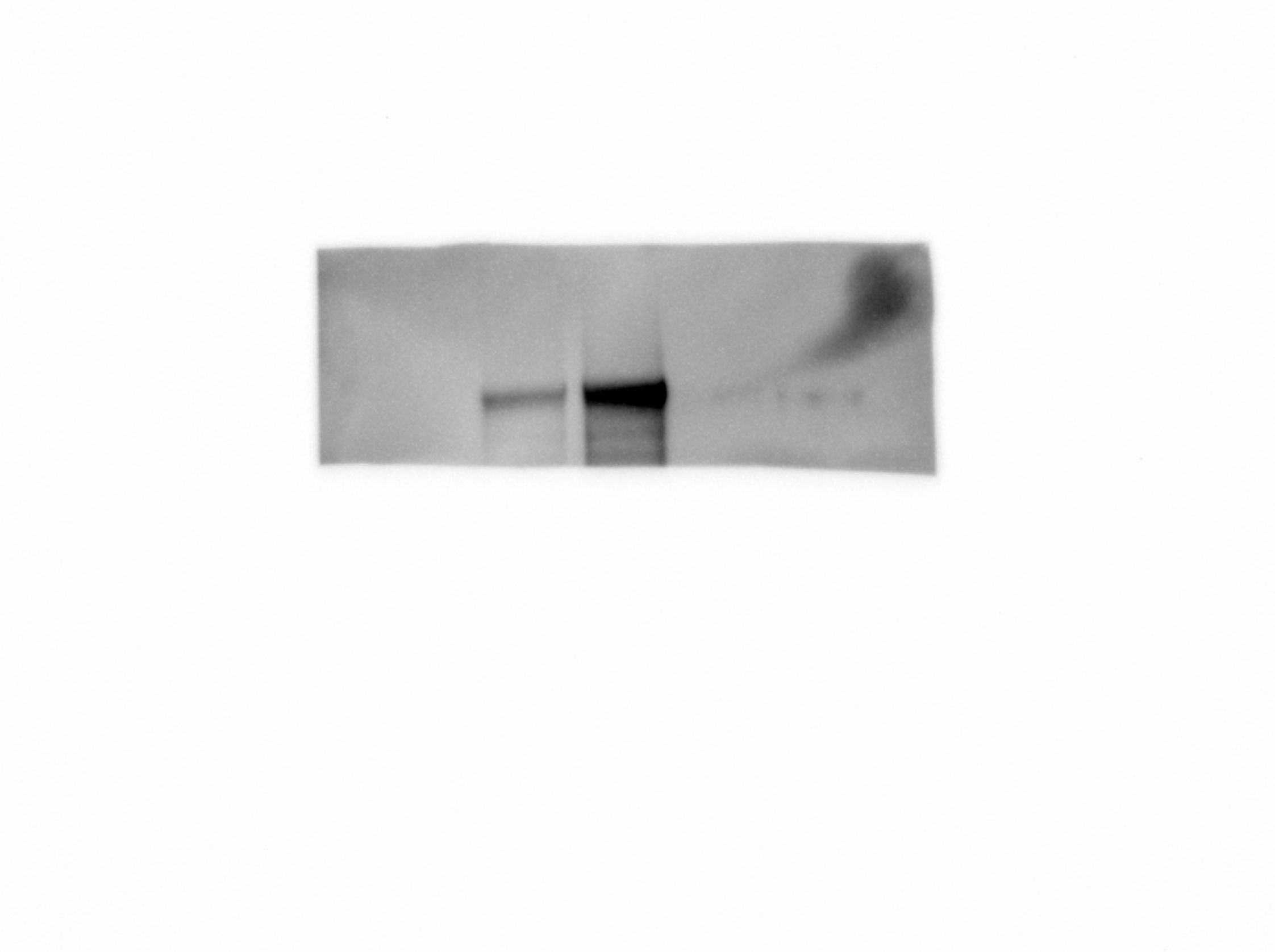

Supplement: Source data 1. [file elife-73982-data1.zip › WesternBlots/Figure4-Source data 1_FN1 (raw).tif]

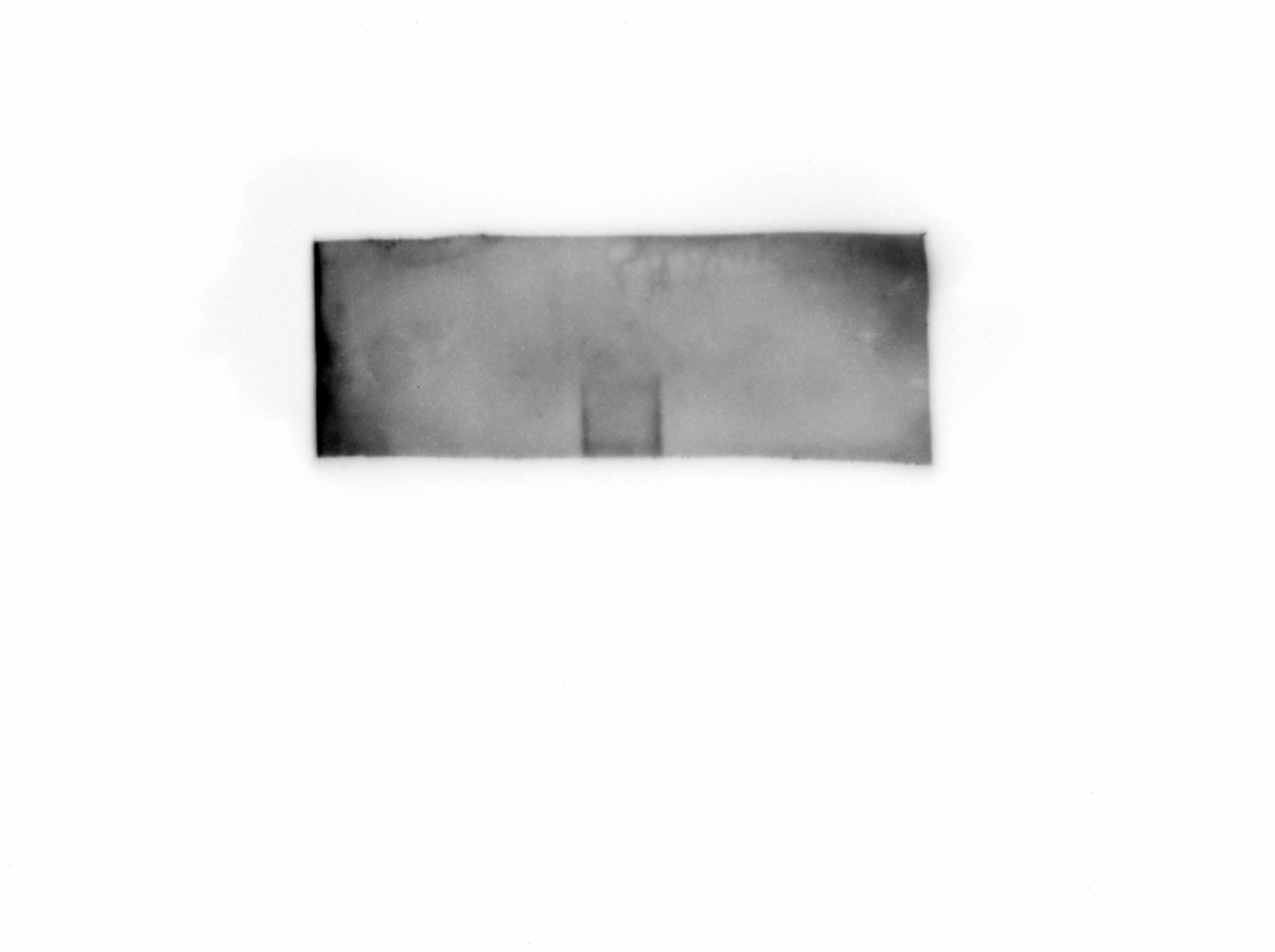

Supplement: Source data 1. [file elife-73982-data1.zip › WesternBlots/Figure4-Source data 1_ABCC1 (raw).tif]

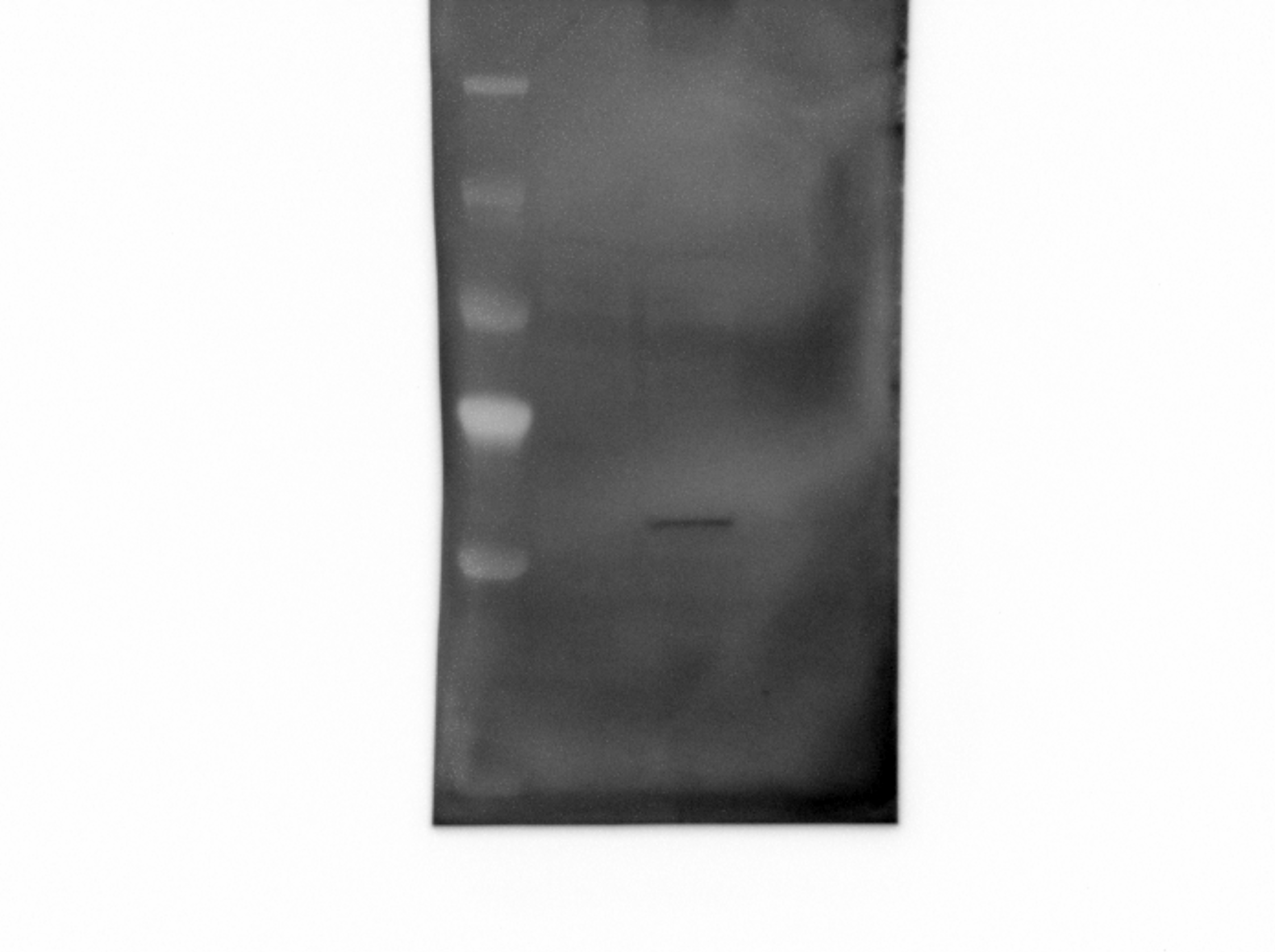

Supplement: Source data 1. [file elife-73982-data1.zip › WesternBlots/Figure3-Source data 1_Vimentin (raw).tif]

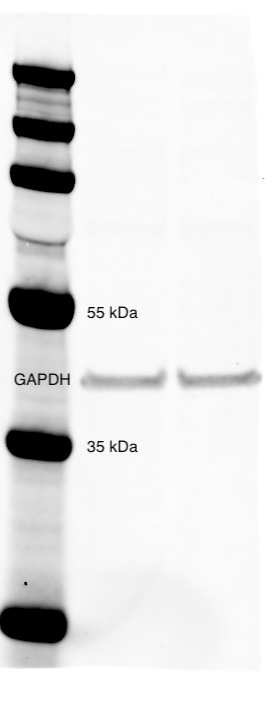

Supplement: Source data 1. [file elife-73982-data1.zip › WesternBlots/Figure3-Source data 1_GAPDH (uncropped).png]

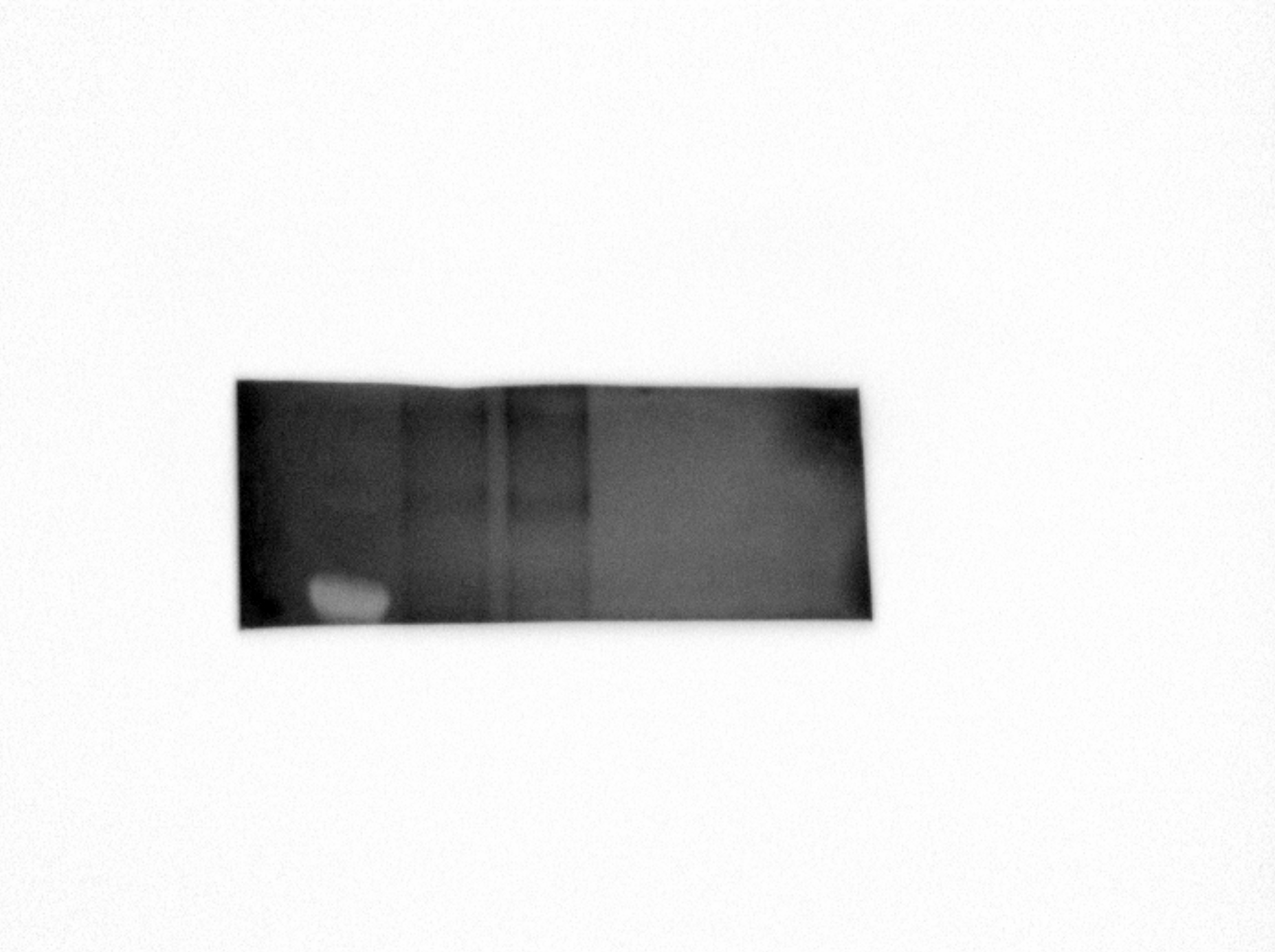

Supplement: Source data 1. [file elife-73982-data1.zip › WesternBlots/Figure5-Source data 1_ITIH4 (raw).tif]

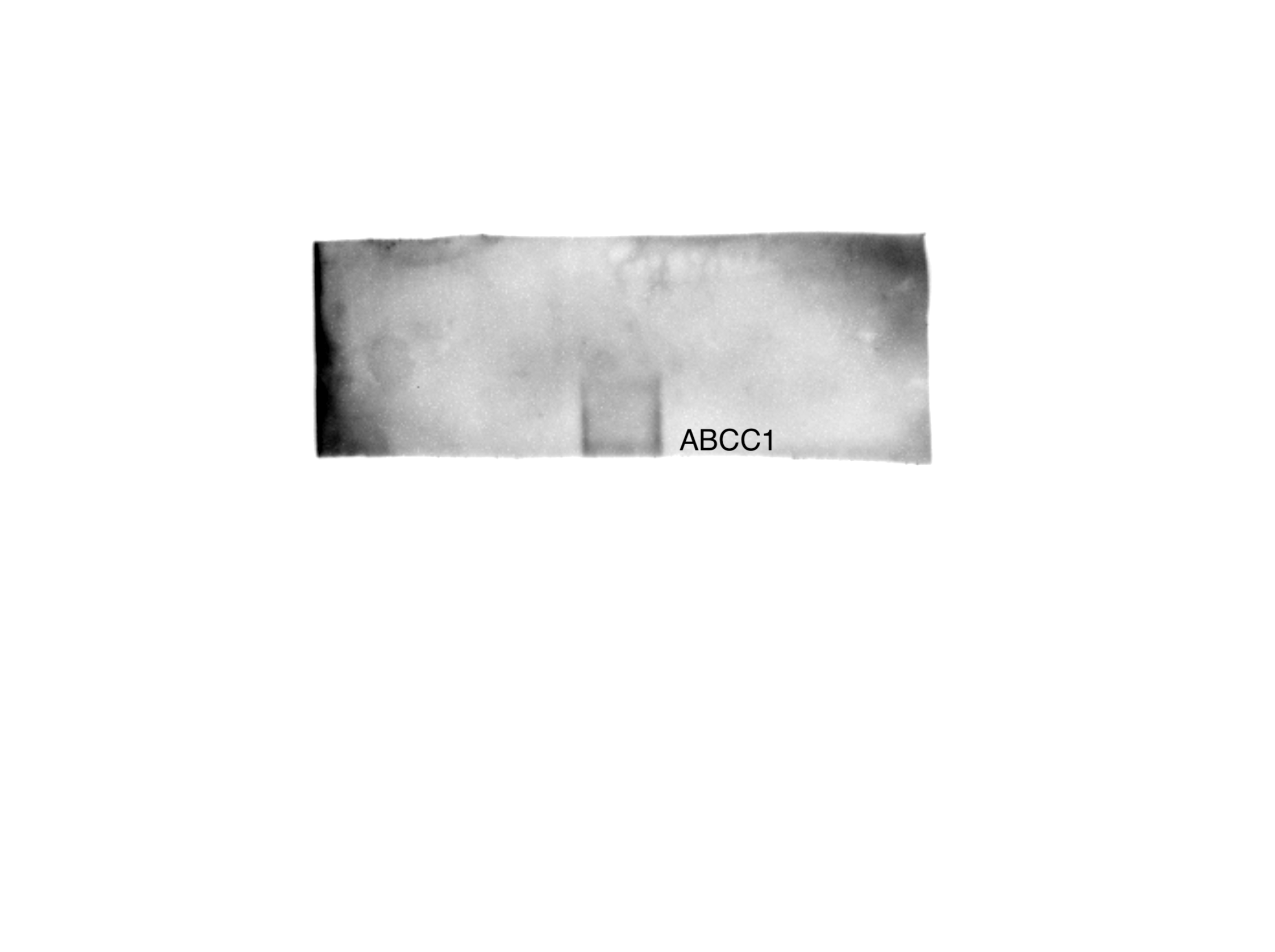

Supplement: Source data 1. [file elife-73982-data1.zip › WesternBlots/Figure4-Source data 1_ABCC1 (uncropped).png]

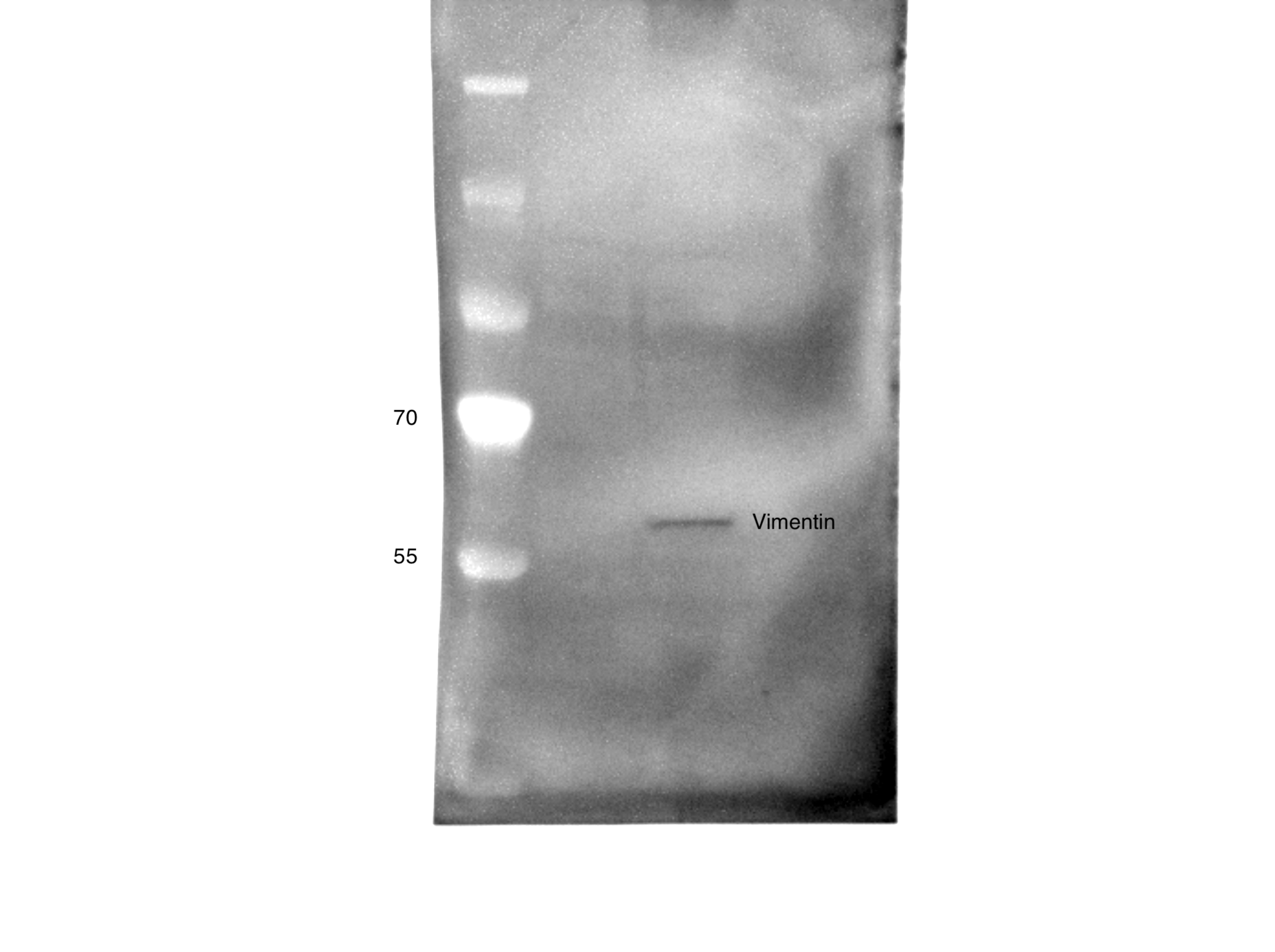

Supplement: Source data 1. [file elife-73982-data1.zip › WesternBlots/Figure3-Source data 1_Vimentin (uncropped).png]

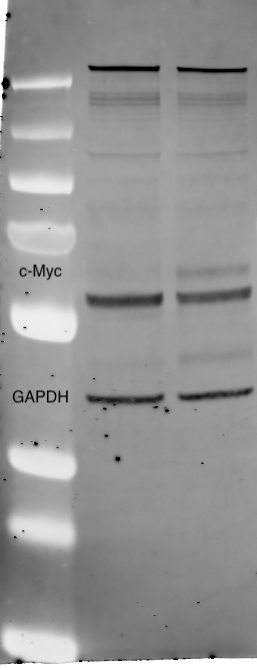

Supplement: Source data 1. [file elife-73982-data1.zip › WesternBlots/Figure3-Source data 1 (uncropped).png]

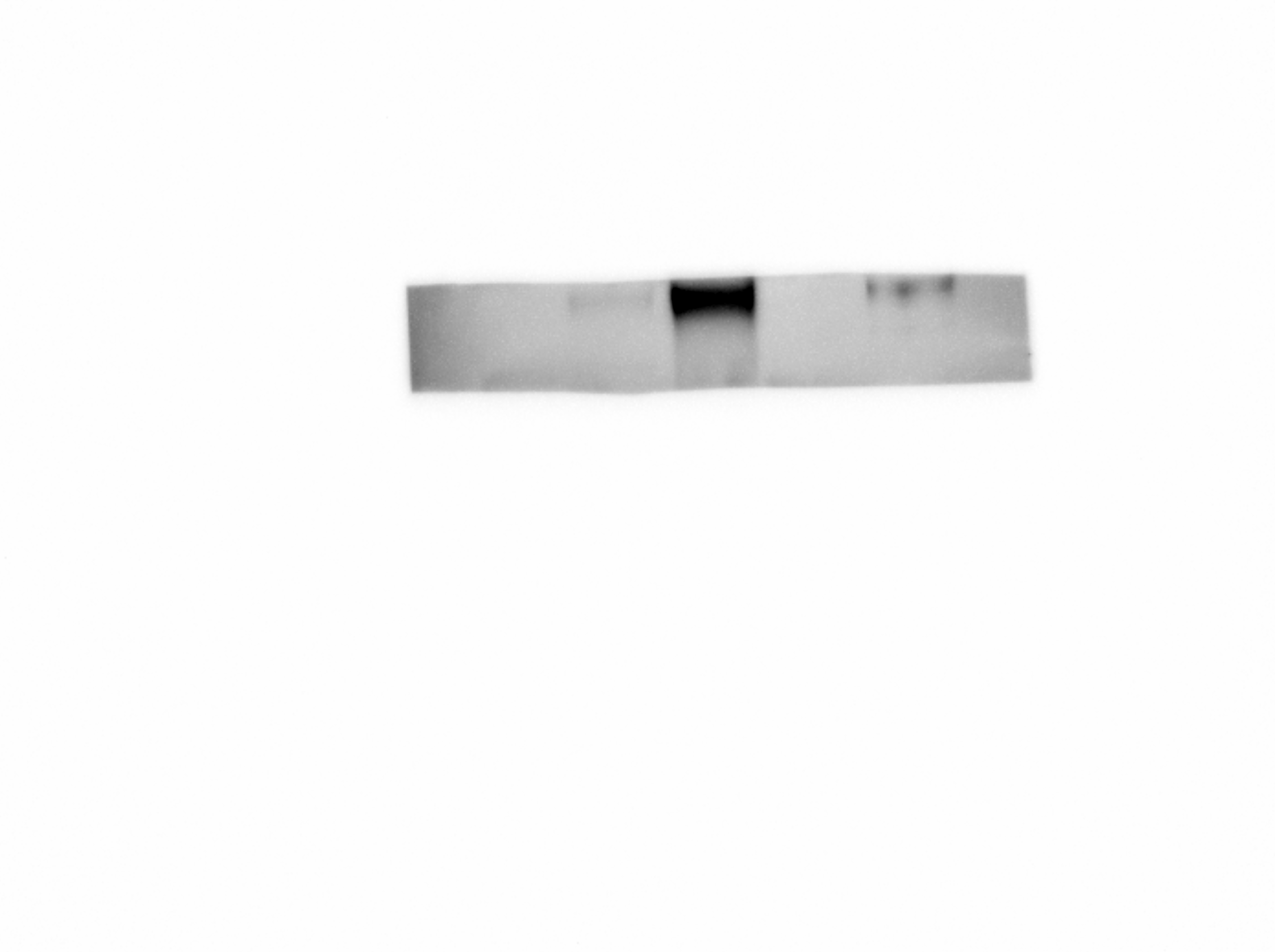

Supplement: Source data 1. [file elife-73982-data1.zip › WesternBlots/Figure4-Source data 1_ANPEP (raw).tif]

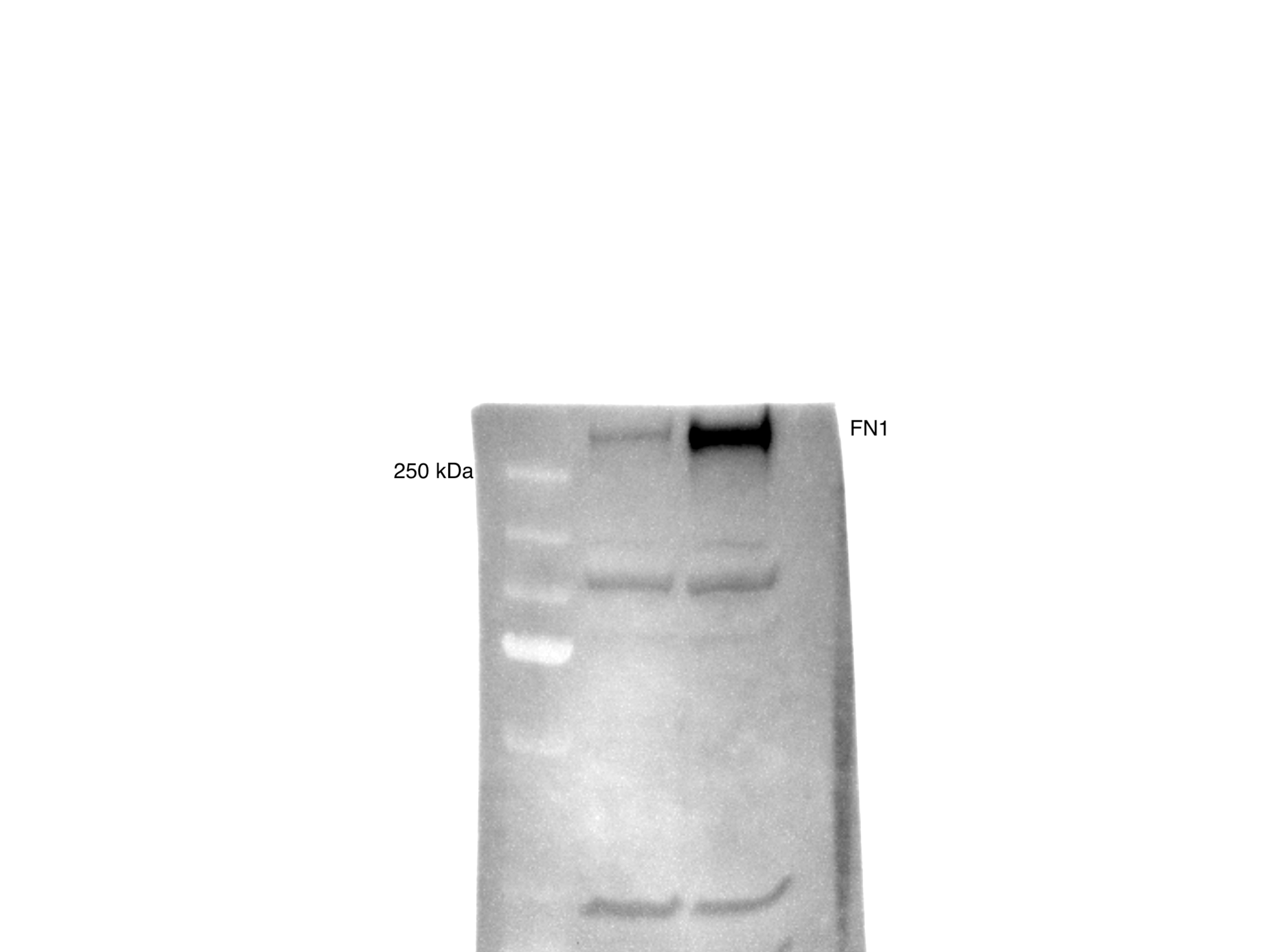

Supplement: Source data 1. [file elife-73982-data1.zip › WesternBlots/Figure3-Source data 1_FN1 (uncropped).png]

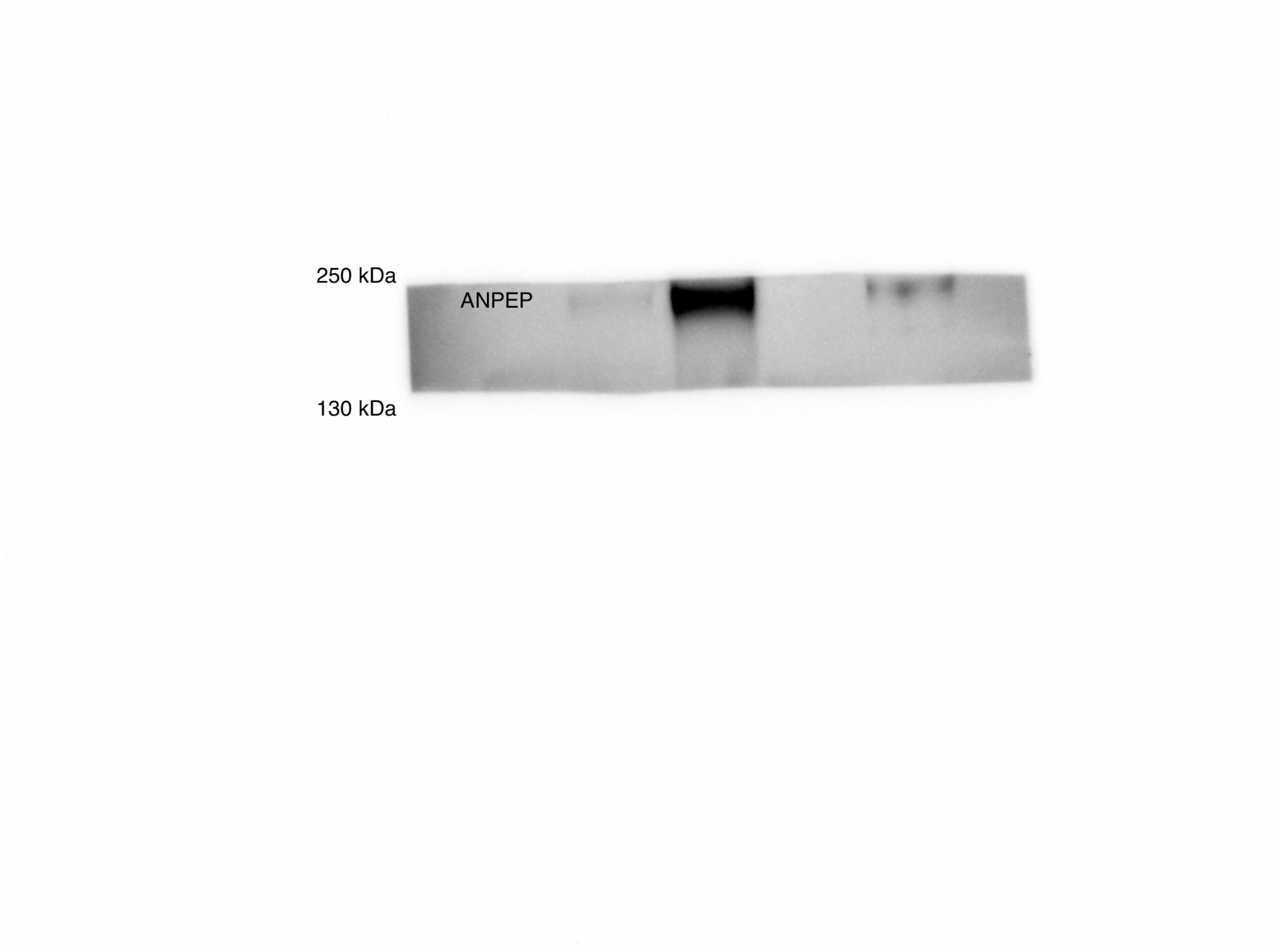

Supplement: Source data 1. [file elife-73982-data1.zip › WesternBlots/Figure4-Source data 1_ANPEP (uncropped).png]

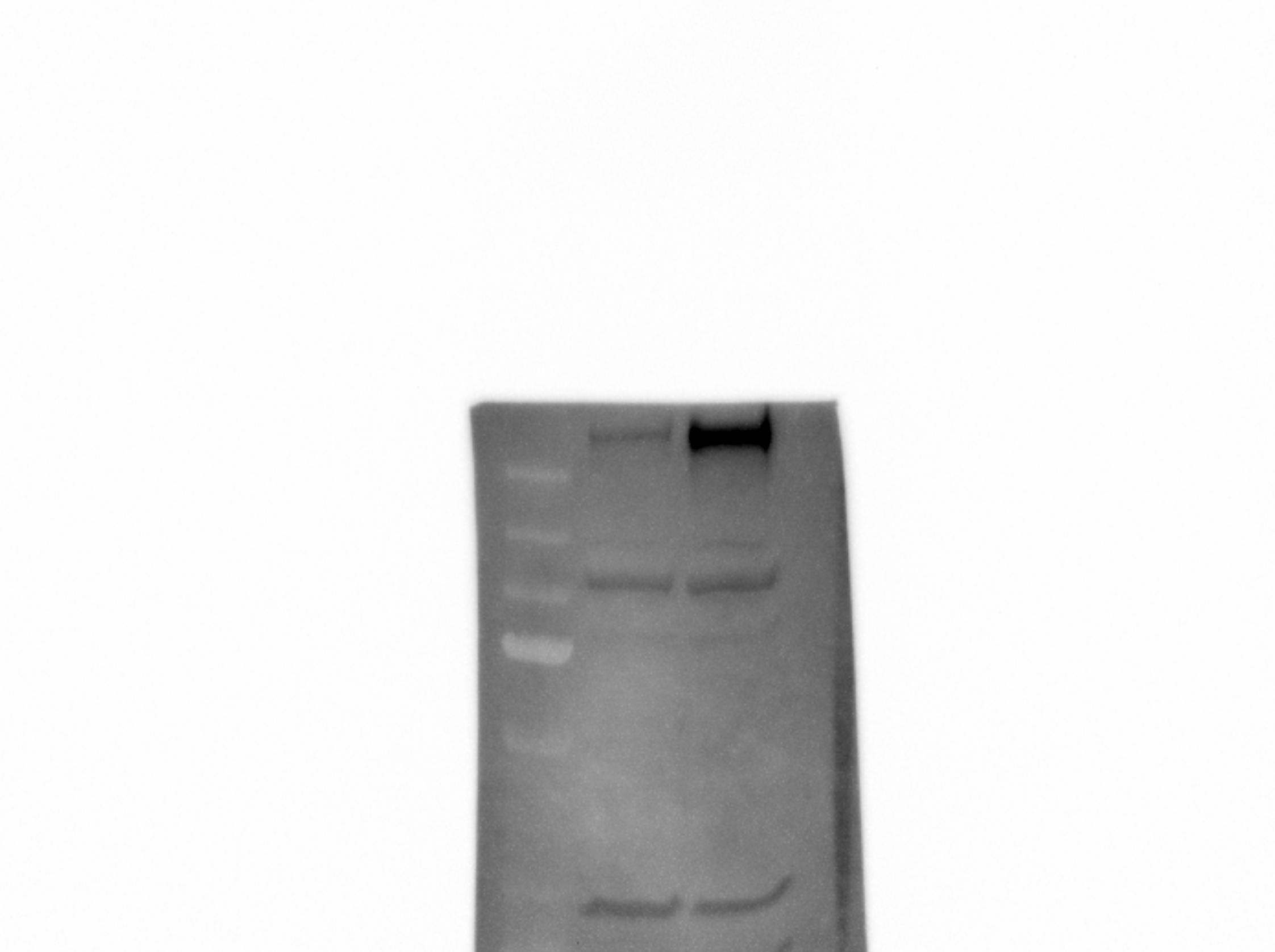

Supplement: Source data 1. [file elife-73982-data1.zip › WesternBlots/Figure3-Source data 1_FN1 (raw).tif]

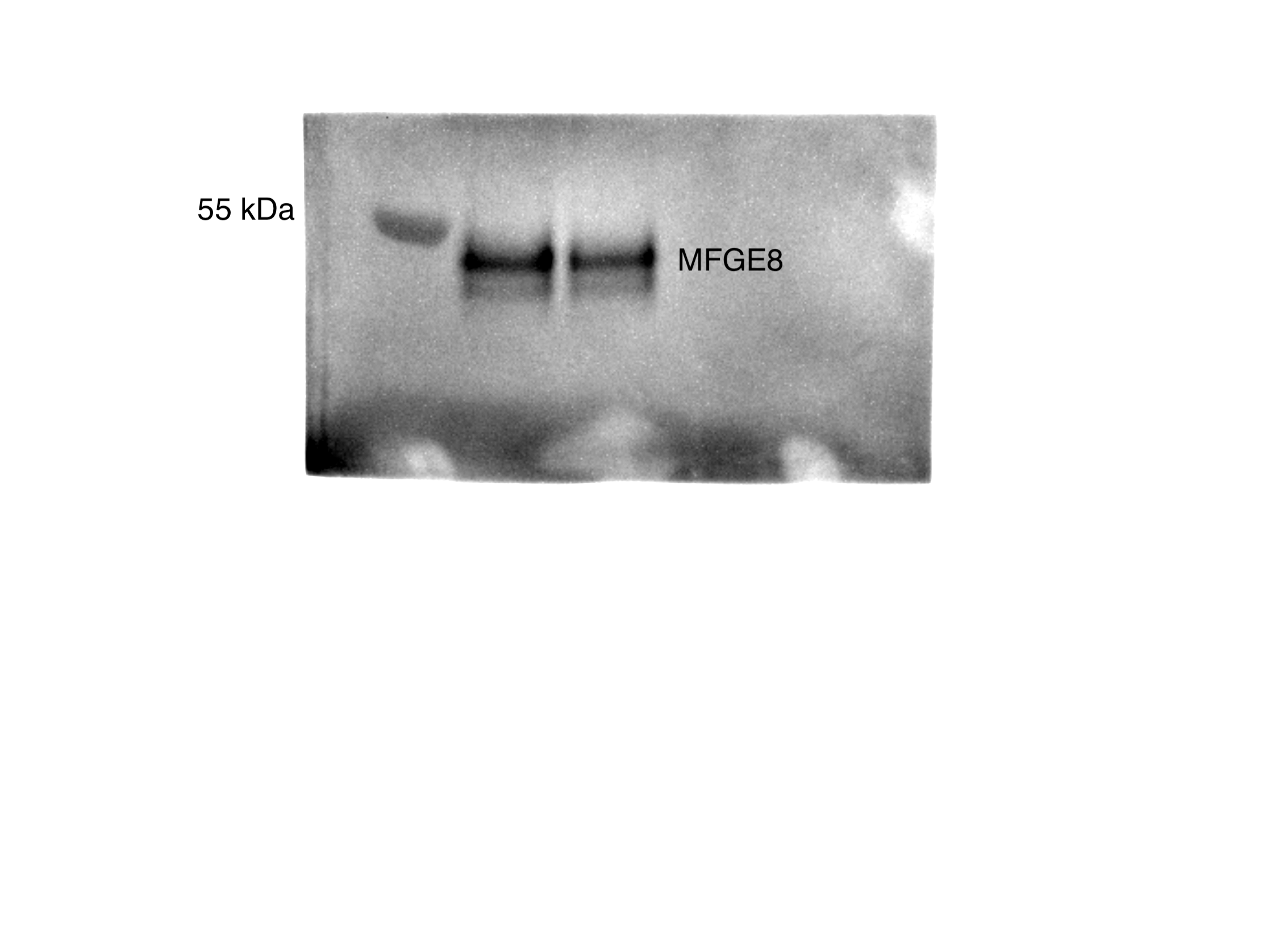

Supplement: Source data 1. [file elife-73982-data1.zip › WesternBlots/Figure5-Source data 1_MFGE8 (uncropped).png]

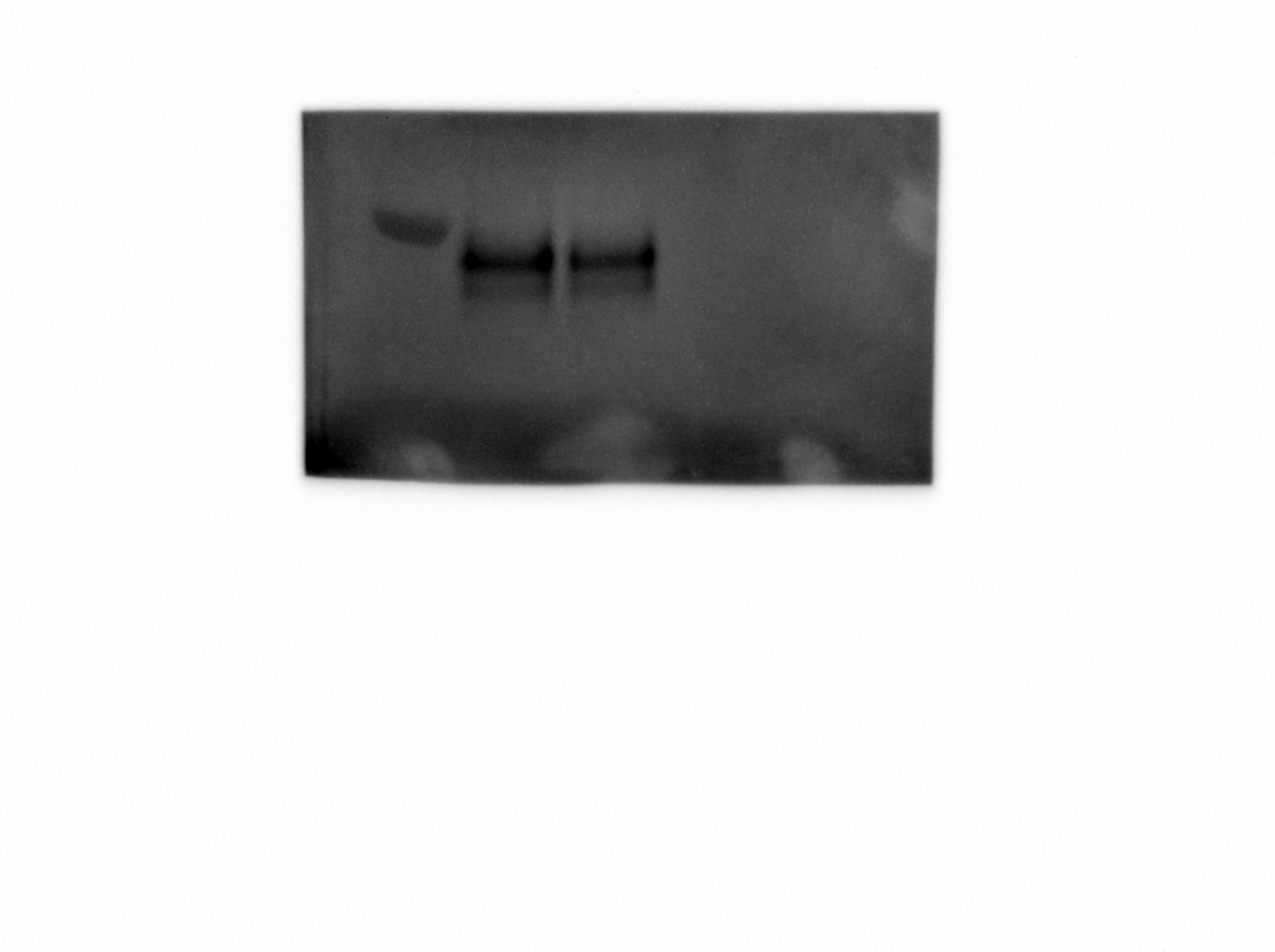

Supplement: Source data 1. [file elife-73982-data1.zip › WesternBlots/Figure5-Source data 1_MFGE8 (raw).tif]

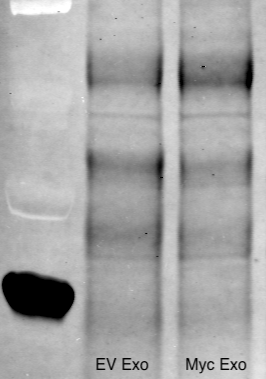

Supplement: Source data 1. [file elife-73982-data1.zip › WesternBlots/Figure4-Source data 1_TotalProtein (uncropped).png]
