# Supplementary material for: Cell-surface tethered promiscuous biotinylators enable comparative small-scale surface proteomic analysis of human extracellular vesicles and cells
Source: eLife. 2022 Mar 8;11:e73982. doi: 10.7554/eLife.73982 (PMC8983049; doi:10.7554/eLife.73982)
Supplement: Transparent reporting form [file elife-73982-transrepform1.docx]

***eLife’s* transparent reporting form**

We encourage authors to provide detailed information *within their submission* to facilitate the interpretation and replication of experiments. Authors can upload supporting documentation to indicate the use of appropriate reporting guidelines for health-related research (see [EQUATOR Network](http://www.equator-network.org/%20)), life science research (see the [BioSharing Information Resource](https://biosharing.org/)), or the [ARRIVE guidelines](http://www.plosbiology.org/article/info:doi/10.1371/journal.pbio.1000412) for reporting work involving animal research. Where applicable, authors should refer to any relevant reporting standards documents in this form.

If you have any questions, please consult our Journal Policies and/or contact us: [editorial@elifesciences.org](mailto:editorial@elifesciences.org).

**Sample-size estimation**

- You should state whether an appropriate sample size was computed when the study was being designed
- You should state the statistical method of sample size computation and any required assumptions
- If no explicit power analysis was used, you should describe how you decided what sample (replicate) size (number) to use

Please outline where this information can be found within the submission (e.g., sections or figure legends), or explain why this information doesn’t apply to your submission:

Replicates numbers were determined from prior experience surrounding the techniques performed and practical considerations. Sample size information is given at end of Methods section and corresponding figure legends.

1. All LFQ-based mass spectrometry was based off of 4 replicate values.

2. Western blot samples were run and quantified 2-3 times and a representative image was displayed in figures. EV blot was run once due to limited sample availability.

3. All other mass spectrometry database searching was based off of two biological replicates.

**Replicates**

- You should report how often each experiment was performed
- You should include a definition of biological versus technical replication
- The data obtained should be provided and sufficient information should be provided to indicate the number of independent biological and/or technical replicates
- If you encountered any outliers, you should describe how these were handled
- Criteria for exclusion/inclusion of data should be clearly stated
- High-throughput sequence data should be uploaded before submission, with a private link for reviewers provided (these are available from both GEO and ArrayExpress)

Please outline where this information can be found within the submission (e.g., sections or figure legends), or explain why this information doesn’t apply to your submission:

General information on replicates is given at end of Methods section and corresponding figure legends.

1. All LFQ-based mass spectrometry was based off of 4 replicate (2 biological, 2 technical) values. Extracellular vesicles (EVs) and cells from different biological replicates were cultured on different days. Desalting, quantification, and LC-MS/MS runs were performed together.

2. Western blot samples were run and quantified 2-3 times and a representative image was displayed in figures. EV blot was run once due to limited sample availability. EVs derived for western blotting were cultured and harvested independently of either biological replicate used for mass spectrometry analysis.

3. All other mass spectrometry database searching was based off of two biological replicates. Biological replicates underwent washing, labeling, and downstream LC-MS/MS preparation separately.

4. No outliers have been excluded in the analysis.

**Statistical reporting**

- Statistical analysis methods should be described and justified
- Raw data should be presented in figures whenever informative to do so (typically when N per group is less than 10)
- For each experiment, you should identify the statistical tests used, exact values of N, definitions of center, methods of multiple test correction, and dispersion and precision measures (e.g., mean, median, SD, SEM, confidence intervals; and, for the major substantive results, a measure of effect size (e.g., Pearson's r, Cohen's d)
- Report exact p-values wherever possible alongside the summary statistics and 95% confidence intervals. These should be reported for all key questions and not only when the p-value is less than 0.05.

Please outline where this information can be found within the submission (e.g., sections or figure legends), or explain why this information doesn’t apply to your submission:

General information on data analysis given at end of Methods section and corresponding figure legends.

Mass spectrometry data was filtered at 1% FDR for both protein and peptide. Proteins were removed if less than 2 unique peptides were identified. Identified proteins were “counted” in the database search results if they were detected across all biological replicates.

Significance for mass spectrometry volcano plots was based off of a standard unpaired Student t test with unequal variances across all four replicates. Reported peak area values represent the averages of all four replicates. Data is provided in datasheet three datasheets titled “Figure 5-source data 2”, “Figure 4-source data 3”, “Figure 3-source data 2”, and “Figure 4-source data 2”.

(For large datasets, or papers with a very large number of statistical tests, you may upload a single table file with tests, Ns, etc., with reference to sections in the manuscript.)

**Group allocation**

- Indicate how samples were allocated into experimental groups (in the case of clinical studies, please specify allocation to treatment method); if randomization was used, please also state if restricted randomization was applied
- Indicate if masking was used during group allocation, data collection and/or data analysis

Please outline where this information can be found within the submission (e.g., sections or figure legends), or explain why this information doesn’t apply to your submission:

NA

**Additional data files (“source data”)**

- We encourage you to upload relevant additional data files, such as numerical data that are represented as a graph in a figure, or as a summary table
- Where provided, these should be in the most useful format, and they can be uploaded as “Source data” files linked to a main figure or table
- Include model definition files including the full list of parameters used
- Include code used for data analysis (e.g., R, MatLab)
- Avoid stating that data files are “available upon request”

Please indicate the figures or tables for which source data files have been provided:

Raw western blot images are made available as a source file titled “Raw_WesternBlot”.

All data from mass spectrometry experiment is provided as source data within the manuscript.

“Figure 3-source data 2” details the quantification results from PEAKS and Perseus for the RWPE-1 +/- Myc cell comparison experiments (N = 4).

“Figure 4-source data 2” details the quantification results from PEAKS and Perseus for the RWPE-1 +/- Myc EV comparison experiments (N = 4).

“Figure 4-source data 3” details the quantification results from MaxQuant and Perseus for the RWPE-1 +/- Myc whole EV experiments (N = 2 for each cell line, which were averaged for the Figure output).

“Figure 5-source data 2” details the quantification results from PEAKS and Perseus for the RWPE-1 +/- Myc EV and cell comparison experiments (N = 4).

“PaTu8902_WGAvsAPEX2_DatabaseSearch” and “KP4_APEX_HRP_Comparison_DatabaseSearch” documents detail results from APEX2 and HRP method comparisons across two different PDAC cell lines (N = 2).

“RWPE_Method_Comparison_DatabaseSearch” outlines the results from the NHS-biotin, biocytin hydrazide, and WGA-HRP comparison experiments performed on RWPE EV and Myc transduced cells (N = 2).

The mass spectrometry proteomics data have been deposited to the ProteomeXchange Consortium via the PRIDE partner repository with the dataset identifier PXD028523.
